# A Comparative Study of Regional Homogeneity of Resting-State fMRI Between the Early-Onset and Late-Onset Recurrent Depression in Adults

**DOI:** 10.3389/fpsyg.2022.849847

**Published:** 2022-04-07

**Authors:** Ji-fei Sun, Li-mei Chen, Jia-kai He, Zhi Wang, Chun-lei Guo, Yue Ma, Yi Luo, De-qiang Gao, Yang Hong, Ji-liang Fang, Feng-quan Xu

**Affiliations:** ^1^Guang’anmen Hospital, China Academy of Chinese Medical Sciences, Beijing, China; ^2^Graduate School of China Academy of Chinese Medical Sciences, Beijing, China; ^3^Institute of Acupuncture and Moxibustion, China Academy of Chinese Medical Sciences, Beijing, China

**Keywords:** major depressive disorder, regional homogeneity, magnetic resonance imaging, early-onset recurrent depression, late-onset recurrent depression

## Abstract

**Background:**

Neurobiological mechanisms underlying the recurrence of major depressive disorder (MDD) at different ages are unclear, and this study used the regional homogeneity (ReHo) index to compare whether there are differences between early onset recurrent depression (EORD) and late onset recurrent depression (LORD).

**Methods:**

Eighteen EORD patients, 18 LORD patients, 18 young healthy controls (HCs), and 18 older HCs were included in the rs-fMRI scans. ReHo observational metrics were used for image analysis and further correlation of differential brain regions with clinical symptoms was analyzed.

**Results:**

ANOVA analysis revealed significant differences between the four groups in ReHo values in the prefrontal, parietal, temporal lobes, and insula. Compared with EORD, the LORD had higher ReHo in the right fusiform gyrus/right middle temporal gyrus, left middle temporal gyrus/left angular gyrus, and right middle temporal gyrus/right angular gyrus, and lower ReHo in the right inferior frontal gyrus/right insula and left superior temporal gyrus/left insula. Compared with young HCs, the EORD had higher ReHo in the right inferior frontal gyrus/right insula, left superior temporal gyrus/left insula, and left rolandic operculum gyrus/left superior temporal gyrus, and lower ReHo in the left inferior parietal lobule, right inferior parietal lobule, and left middle temporal gyrus/left angular gyrus. Compared with old HCs, the LORD had higher ReHo in the right fusiform gyrus/right middle temporal gyrus, right middle temporal gyrus/right angular gyrus, and left rolandic operculum gyrus/left superior temporal gyrus, and lower ReHo in the right inferior frontal gyrus/right insula. ReHo in the right inferior frontal gyrus/right insula of patients with LORD was negatively correlated with the severity of 17-item Hamilton Rating Scale for Depression (HAMD-17) scores (*r* = −0.5778, *p* = 0.0120).

**Conclusion:**

Adult EORD and LORD patients of different ages have abnormal neuronal functional activity in some brain regions, with differences closely related to the default mode network (DMN) and the salience network (SN), and patients of each age group exhibit ReHo abnormalities relative to matched HCs.

**Clinical Trial Registration:**

[http://www.chictr.org.cn/], [ChiCTR1800014277].

## Introduction

Major depressive disorder (MDD) is a common clinical psychiatric disorder that is characterized by depressed mood, slowed thinking, reduced interest, and diminished cognitive function as the main clinical manifestations, and is one of the main causes of disability (Rotenstein et al., 2016; Cladder-Micus et al., 2018). It is estimated that MDD will become the second leading cause of disability in the world (Ferrari et al., 2013). Approximately 25% of MDD patients relapse within 6 months of discharge (Harlev et al., 2021), approximately one-third of MDD patients are prone to relapse within a year (Cosci et al., 2020), and the severity of MDD increases with the number of depressive relapses (Harlev et al., 2021). There is a growing awareness that the challenge of depression is relapse prevention rather than recovery (Fava et al., 2017). Therefore, understanding the pathogenesis of recurrent depression is of great importance for clinical work.

Previous studies have found that patients with recurrent depression at different ages have different clinical features (Driscoll et al., 2005; Hammen et al., 2008; Sung et al., 2013). Early onset recurrent depression (EORD) is associated with a higher risk of life-long persistent depression, a higher risk of suicide, worse social cognitive function, and greater anxiety than late onset recurrent depression (LORD) (Hammen et al., 2008; Sung et al., 2013). And LORD is also associated with more cognitive dysfunction than EORD (Driscoll et al., 2005). A study has shown that EORD has a greater risk of relapse than LORD (Cassano et al., 1993). Therefore, recurrent depression in different age groups may be associated with different neuropathological mechanisms.

Moreover, recurrent depression is more heritable and increases the risk of depression in offspring (Burcusa and Iacono, 2007; Benjet et al., 2020; Jaffee et al., 2021). For example, one study found that adolescent children whose parents had recurrent depression had a 4.21-fold greater probability of reaching MDD than adolescents whose parents were never depressed (Jaffee et al., 2021). A meta analysis showed a degree of specificity in different subtypes of depression, and understanding their specificity can be a guide to clinical treatment (Harald and Gordon, 2012).

However, the clinical characteristics of EORD and LORD depend on the different cut-off ages (Cassano et al., 1993; Driscoll et al., 2005; Hammen et al., 2008). An age-specific study of recurrent depression in adolescents showed that EORD recurred between 15 and 20 years of age, and LORD after 20 years of age (Hammen et al., 2008). A study of recurrent depression in older adults showed that the age of EORD was defined as before 59 years of age, while LORD was defined as after 60 years of age (Driscoll et al., 2005). Another study defined the age of EORD before 44 years of age and LORD after 45 years of age (Cassano et al., 1993). The above studies suggest that the biological evidence for the age limits of EORD and LORD is unclear and there is no unified consensus on age classification criteria. In this study, the age of EORD was defined as 18 to 29 years and LORD was defined from 30 to 45 years based on previous studies to avoid the effect of (>45 years) cerebrovascular disease (Shen et al., 2017).

In recent years, with the development of neuroimaging technology, resting-state functional MRI (rs-MRI) has been widely used in insomnia (Li et al., 2019), schizophrenia (Huang et al., 2020), autism (Shi et al., 2020), and other psychiatric disorders. Regional homogeneity (ReHo) is an important indicator for the study of rs-fMRI. ReHo can reflect the synchronization of brain functional activity state in localized regions of whole-brain voxels and is used to assess the level of coordination of neural activity in local brain regions (Zang et al., 2004). In addition, the ReHo indicator has been used to study changes in neuronal functional activity in depressive subtypes of the brain, and abnormalities have been found in brain regions such as the frontal lobe, precuneus, and insula (Chen et al., 2012; Shen et al., 2017; Zhang et al., 2021). The ReHo values of these abnormal brain regions are closely correlated with the default mode network (DMN) and the salience network (SN). The correlation of ReHo values with abnormal brain regions in subtypes of depression is useful for understanding the degree of severity of the disease and neuroimaging markers (Shen et al., 2017; Liu et al., 2021). Previous studies have also found that differences in brain function across subtypes of depression are associated with abnormalities in DMN and SN (Li et al., 2017; Cai et al., 2021). However, there is a lack of neuroimaging studies on the differences between EORD and LORD, and the differences in regional neural activity between EROD and LORD are unclear.

In this study, we focused on the differences in local brain functional activity between EORD (18–29 years) and LORD (30–45 years) and whether there was a correlation between the depression group and clinical symptoms. Based on previous studies of age differences in subtypes of depression, we hypothesized that (1) differences in local brain functional activity in EORD and LORD may be closely related to the DMN and SN. (2) Increased or decreased local brain function of ReHo in EORD and LORD may be associated with clinical depressive symptoms.

## Materials and Methods

### Subjects

A total of 36 patients (18 with EORD and 18 with LORD) were diagnosed with recurrent depression from the Department of Psychosomatic Medicine, Guang’anmen Hospital, China Academy of Chinese Medical Sciences, the Department of Psychiatry, Beijing First Hospital of Integrative Medicine, the Department of Psychiatry, Yuquan Hospital, Qinghua University, and the Department of Psychiatry, Xuanwu Hospital, Capital Medical University. The inclusion criteria were as follows: (1) According to the diagnostic criteria for depression in the Diagnostic and Statistical Manual of Mental Disorders (DSM-V) of the American Psychiatric Association, all patients were diagnosed by two experienced psychiatrists; (2) All patients recurred after antidepressant treatment, relapsed between the ages of 18 and 45 years, and were off medication for at least 4 weeks prior to admission; (3) 17-item Hamilton Rating Scale for Depression (HAMD-17) score > 17; and (4) right-handedness. 36 gender- and age-matched healthy controls (HCs; 25 females and 11 males) were also included in the HCs: (1) HAMD-17 score < 7; (2) right-handedness; and (3) no history of mental illness.

Exclusion criteria of patients and HCs include the following items: (1) suffering from serious mental illness and other diseases such as cardiovascular and cerebrovascular; (2) with a history of drug and alcohol abuse; (3) had any MRI contraindications, such as heart pacemaker, metal fixed false teeth, or severe claustrophobia; and (4) were pregnant or lactating.

### Clinical Materials and Subgroups

Data were collected for the study population including gender, age, education level, frequency of recurrence, and duration of disease. Subjects were assessed for depressive symptoms by an experienced psychiatrist using the HAMD-17 scale. Based on previous studies on age division of first-episode depression to avoid the influence of cerebrovascular diseases (Shen et al., 2016, 2017), we initially divided all recurrent depression patients with recurrent depression into EORD group (18–29 years) and LORD group (30–45 years). The HCs were also matched corresponding to two subgroups: young HCs (18–29 years) and old HCs (30–45 years).

### Scan Acquisition

All subjects in this study underwent data acquisition using a Magneton Skyra 3.0 T MRI scanner (Siemens, Germany). Subjects were informed prior to the scan to remain awake during the scan and to avoid active thinking activities. During the scan, the subject is required to use earplugs and wear noise-canceling headphones, use a hood to immobilize the head, and lie flat on the examination bed. The scanning procedure contains a localizer, a high resolution three-dimensional T1-weighted imaging (3D-T1WI), and a blood oxygenation level-dependent fMRI (BOLD-fMRI).

The scanning parameters were as follows: 3D-T1WI: time repetition (TR)/time echo (TE) = 2500/2.98 ms, flip angle = 7°, matrix = 64 × 64, field of view (FOV) = 256 mm × 256 mm, slice thickness = 1 mm, slice number = 48, slices =192, scanning time 6 min 3 s; BOLD-fMRI: TR/TE = 2000/30 ms, flip angle = 90°, matrix 64 × 64, field of view = 240 mm × 240 mm, slice number = 43, slice thickness/spacing = 3.0/1.0 mm, and 200 volumes were obtained, scanning time 6 min 40 s.

### fMRI Data Analysis

#### fMRI Data Preprocessing

The rs-fMRI data were pre-processed using Data Processing Assistant for Resting-State fMRI (DPARSF) software (DPABI5.0; Chao-Gan and Yu-Feng, 2010) in MATLAB (Mathworks, Inc., Natick, MA, United States).^1^ First, image format conversion was performed to convert the raw data DICOM format to NIFTI format. Next, the first 10 time points were excluded. Then, slice timing, realign, was performed to remove subjects with head movement translations exceeding 2.0 mm and rotations exceeding 2.0°. Spatial normalization was performed by normalizing the functional images to Montreal Neurological Institute (MNI) space. Linear regression was performed on the covariates of head movement, white matter (WM), and cerebrospinal fluid (CSF) signals to reduce the effects. Finally, we removed the linear drift and set the filter to 0.01–0.08 Hz to reduce the effect of noise.

#### ReHo Analysis

The pre-processed data images were analyzed using DPARSF software, and ReHo was calculated by Kendall correlation coefficient (Kendall & Gibbons, 1990), based on voxels to calculate the synchronization of the time series of a given voxel with the time series changes of its 26 adjacent voxels, and a ReHo map was obtained for each subject. The ReHo map of each subject was divided by the whole-brain mean ReHo value to obtain a normalized ReHo map. Finally, the smReHo maps were obtained by smoothing using a 6 mm × 6 mm × 6 mm Gaussian smoothing kernel for subsequent statistical analysis.

### Statistical Analysis

#### Clinical Data Analysis

The clinical data were analyzed with SPSS 23.0 statistics software (IBM Corp, Somers, New York, United States). One-way analysis of variance (ANOVA) was used to compare age and education level between the four groups, and the chi-square test was used to compare gender. The duration of illness, frequency of recurrence, and HAMD-17 scores were compared between patient groups using a two-sample *t*-test. *p* < 0.05 was statistically significant.

#### fMRI Data Analysis

Image data statistics were analyzed using the DPARSF toolbox, and a voxel-based one-way ANOVA was performed to compare whole-brain ReHo maps of the four groups. Gender, age, education level, and framewise displacement (FD) metric (derived from Jenkinson’s formula) of the four groups of subjects were used as covariates, brain areas with ReHo differences between the four groups were corrected for Gaussian random fields (GRF), and corrected cluster levels were settled as *p* < 0.05 and threshold voxel levels *p* < 0.005 were defined as statistically different. The time series means of the peak voxel ReHo values of the four contrasting brain regions were extracted, and *post-hoc* two-sample *t*-tests were performed on the ReHo values between the paired groups (EORD *vs.* young HCs, LORD *vs.* old HCs, LORD *vs.* EORD) using SPSS 23.0 software, and the results were Bonferroni corrected (*p* < 0.01).

In order to verify the relationship between ReHo values and clinical symptoms, Pearson correlation analysis was performed by extracting ReHo values of abnormal brain regions and HAMD-17 scores in patient groups, separately, and *p* < 0.05 was statistically significant.

## Results

### Characteristics of Research Samples

A total of 18 patients with EROD, 18 patients with LORD, 18 young HCs, and 18 old HCs met the study criteria. There were no statistical differences between the MDD group and the HCs and in terms of gender and years of education, and there were statistical differences in age comparisons between the four groups. There were no statistical differences in disease duration, frequency of recurrence, and HAMD-17 scores between the two MDD groups (Table 1).

**Table 1:** Demographic and clinical characteristics of the study participants.

Variables (mean ± SD)	HCs		MDD		*t(F)/χ* ^2^	*p*
Age (years)	31.61 ± 7.01		31.58 ± 7.69		0.016	0.987^a^
Gender (male/female)	11/25		10/26		0.670	0.795^b^
Years of education	15.80 ± 3.90		15.02 ± 2.96		0.952	0.344^a^
HAMD-17 score	1.97 ± 0.99		23.61 ± 3.31		−37.501	0.000^a^
	**Young HCs**	**Old HCs**	**EORD**	**LORD**		
Age (years)	25.16 ± 3.36	38.00 ± 4.85	25.94 ± 2.31	37.27 ± 5.28	51.594	0.000^c^
Gender (male/female)	5/13	6/12	4/14	6/12	0.739	0.864^d^
Years of Education	16.72 ± 2.46	13.77 ± 4.27	15.38 ± 2.11	14.66 ± 3.64	2.643	0.056^e^
Frequency of recurrence			1.33 ± 0.59	1.44 ± 0.61	−0.551	0.585^a^
Illness duration (months)			19.77 ± 12.37	23.16 ± 15.64	−0.721	0.476^a^
HAMD-17 score	1.94 ± 1.05	2.00 ± 0.97	23.72 ± 3.23	23.38 ± 3.53	−0.397	0.694^f^

*HCs, healthy controls; MDD, major depressive disorder; EORD, early onset recurrent depression; LORD, late onset recurrent depression; and HAMD-17, 17-item Hamilton Rating Scale for Depression.*

a Value of *p obtained by two-sample t-test.*

b Value of *p for gender distribution in the two groups was obtained by chi-square test.*

c Value of *p were obtained by one-way analysis of variance tests. Post-hoc t-test: p = 0.964(EORD* vs. *Young HCs), p = 0.999(LORD* vs. *Old HCs).*

d Value of *p of gender distribution among the four groups were obtained by chi-square test: p = 0.717(EORD* vs. *Young HCs), p = 0.457(LORD* vs. *Old HCs).*

e *The* values of *p were obtained by one-way analysis of variance tests. Post-hoc t-test: p = 0.222(EORD* vs. *Young HCs), p = 0.507(LORD* vs. *Old HCs).*

f Values of *p were obtained by two-sample t-test p = 0.694 (EORD* vs. *LORD).*

### ReHo: Group Differences

**Figure 1:**
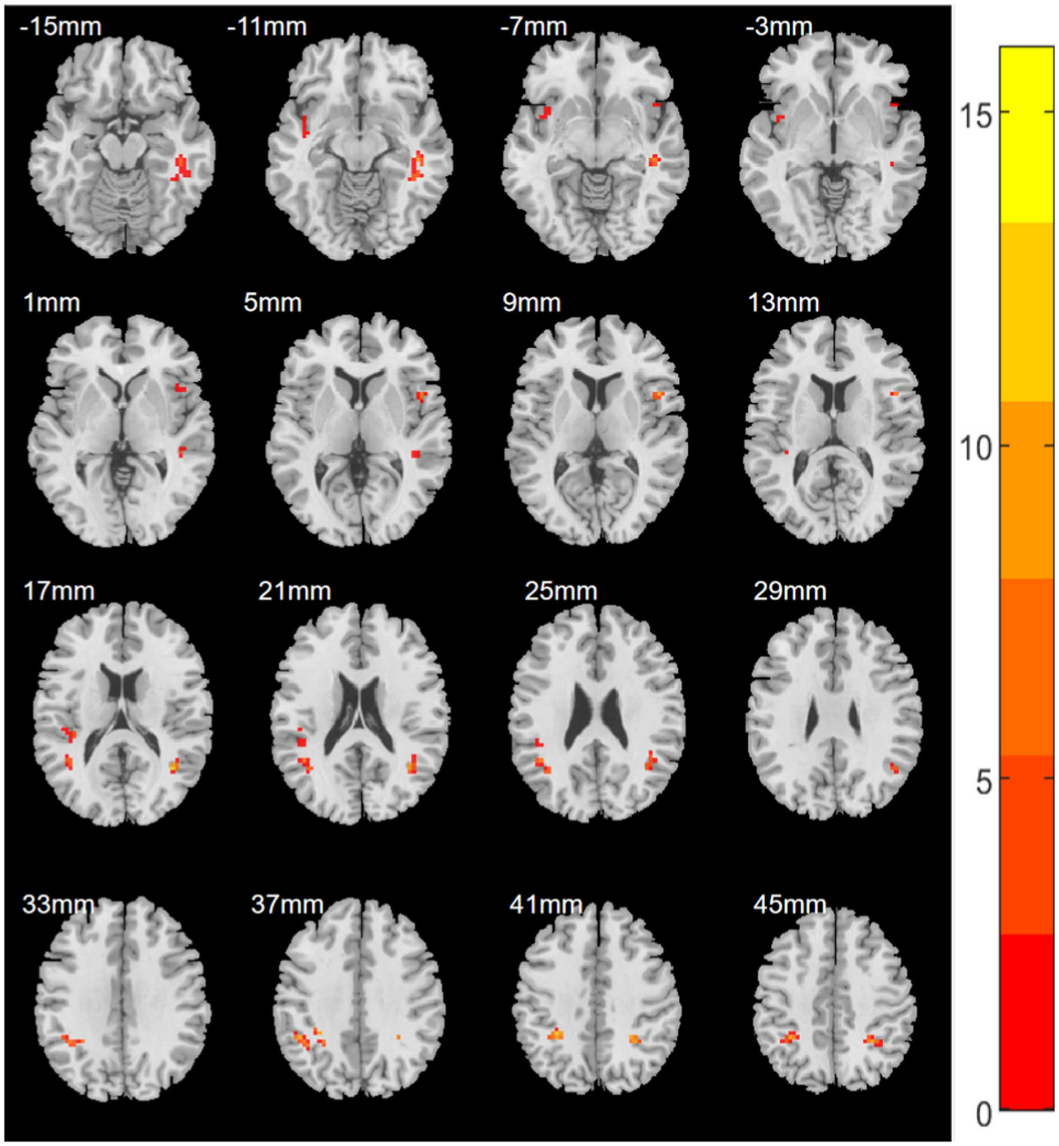
Statistical maps showing ANOVA results for ReHo abnormalities between EORD or LORD patients and HCs (GRF corrected). Color bar indicates red to yellow showing enhancement of ReHo values.

A one-way ANOVA was performed on the ReHo values of the four groups using age, gender, years of education, and mean frame displacement as covariates. ReHo was found to be significantly significant difference in the right inferior frontal gyrus/right insula, left superior temporal gyrus/left insula, left inferior parietal lobule, right inferior parietal lobule, right fusiform gyrus/right middle temporal gyrus, left middle temporal gyrus/left angular gyrus, right middle temporal gyrus/right angular gyrus, and left rolandic operculum gyrus/left superior temporal gyrus significant differences between superior gyrus groups (Table 2; Figure 1).

**Table 2:** One-way ANOVA between the four groups for differences in ReHo values Brain areas.

Brain regions	Side	Peak coordinates (MNI)			Cluster size	*F*-values
		X	Y	Z		
Inferior frontal gyrus/insula	R	48	12	9	26	12.06
Superior temporal gyrus/insula	L	−42	−6	−9	24	7.176
Inferior parietal lobule	L	−27	−42	39	75	15.99
Inferior parietal lobule	R	21	−42	54	74	14.1
Fusiform gyrus/middle temporal gyrus	R	45	−27	−9	67	14.4
Middle temporal gyrus/angular gyrus	L	−42	−51	21	27	9.305
Middle temporal gyrus/angular gyrus	R	39	−57	18	33	13.215
Rolandic operculum gyrus/superior temporal gyrus	L	−36	−30	18	23	7.576

*F is the statistical value of the difference in peak voxel ReHo between the EORD and LORD groups and HCs. p < 0.05, GRF corrected, p < 0.005, cluster size > 10. Abnormal ReHo in EORD patients VS young HC.*

**Figure 2:**
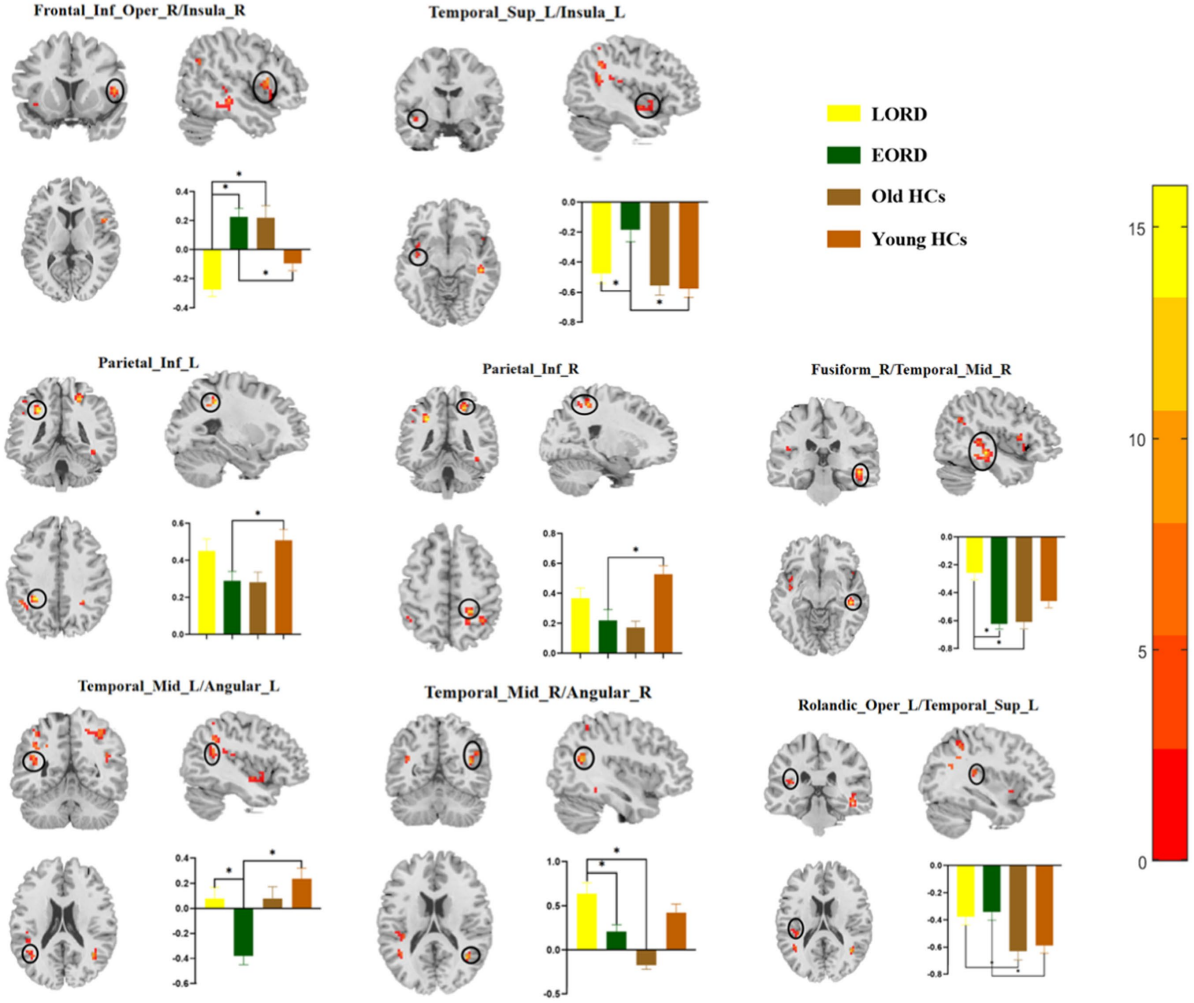
*Post-hoc* two-sample *t*-tests (Bonferroni corrected) comparison showing ReHo values differences at peak voxel between each pair group (EORD vs. young HCs, LORD vs. old HCs, and LORD vs. EORD). EORD, early onset recurrent depression; LORD, late onset recurrent depression; young HCs, young healthy controls; and old HCs, old healthy controls. Frontal_Inf_Oper_R/Insula_R = right inferior frontal gyrus/right insula; Temporal_Sup_L/Insula_L = left superior temporal gyrus/left insula; Parietal_Inf_L = left inferior parietal lobule; Parietal_Inf_R = right inferior parietal lobule; Fusiform_R/Temporal_Mid_R = right fusiform gyrus/right middle temporal gyrus; Temporal_Mid_L/Angular_L = left middle temporal gyrus/left angular gyrus; Temporal_Mid_R/Angular_R = right middle temporal gyrus/right angular gyrus; and Rolandic_Oper_L/Temporal_Sup_L = left rolandic operculum gyrus/left superior temporal gyrus.

Compared with young HCs, the EORD had higher ReHo in the right inferior insular frontal gyrus/right insula, left superior temporal gyrus/left insula, and left rolandic operculum gyrus/superior temporal gyrus, and lower ReHo values in the left inferior parietal lobule, right inferior parietal lobule, and left middle temporal gyrus/left angular gyrus (Figure 2).

### Abnormal ReHo in LORD Patients *vs* Old HC

Compared with old HCs, the LORD had higher ReHo in the right fusiform gyrus/right middle temporal gyrus, right middle temporal gyrus/right angular gyrus, left rolandic operculum gyrus/left superior temporal gyrus, and lower ReHo values in the right inferior frontal gyrus/right insula (Figure 2).

### Abnormal ReHo in LORD Patients *vs* EORD

Compared with EORD, the LORD group had higher ReHo in the right fusiform gyrus/right middle temporal gyrus, left middle temporal gyrus/left angular gyrus, and right middle temporal gyrus/right angular gyrus, and lower ReHo in the right inferior frontal gyrus/right insula and left superior temporal gyrus/left insula (Figure 2).

### Correlations Between the Abnormal ReHo and Depressive Symptoms in the Patients

**Figure 3:**
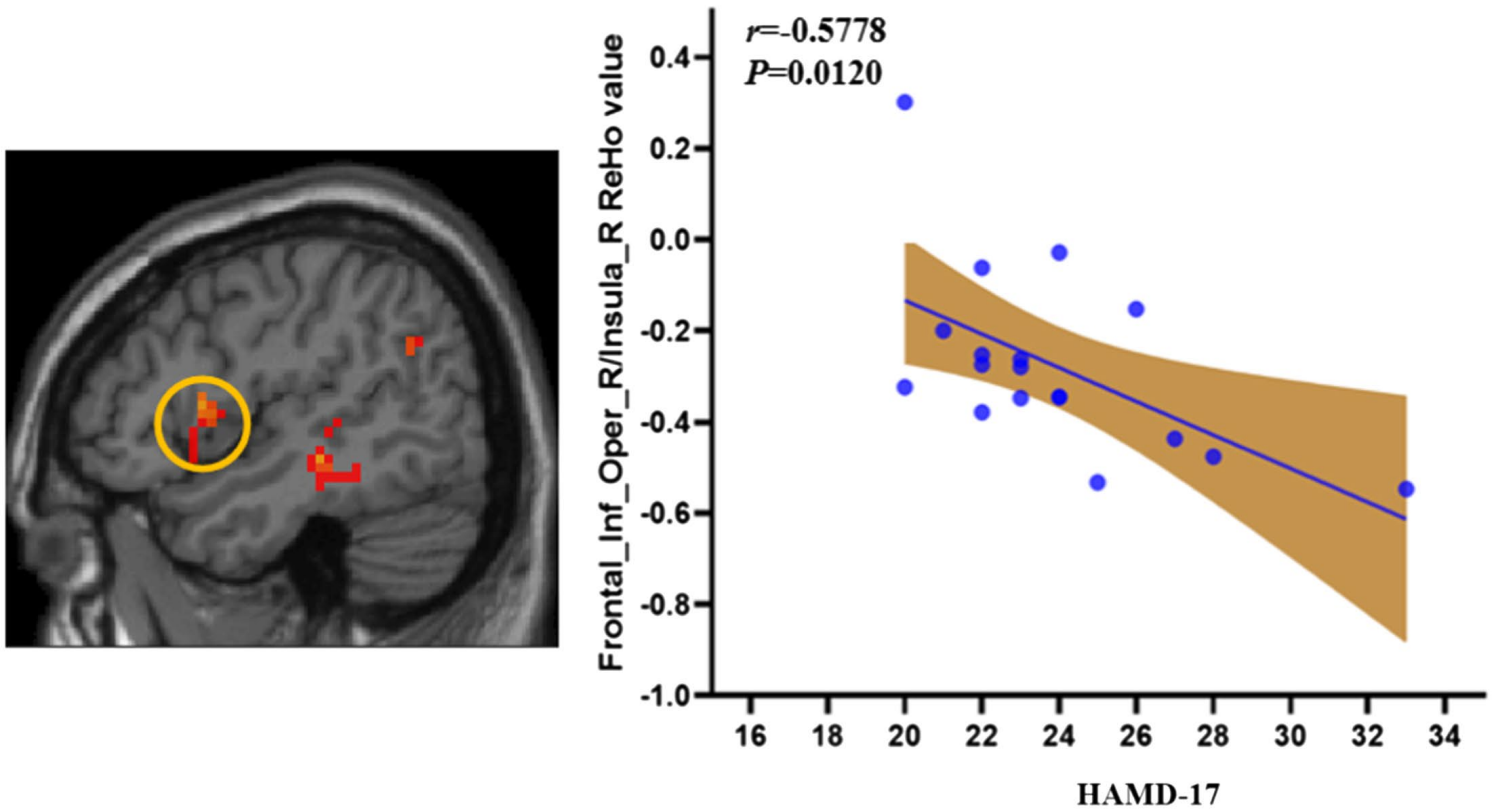
The correlation of the ReHo and HDRS symptom in LORD patients. LORD, late onset recurrent depression.

To test whether there was a relationship between the severity of depressive symptoms and abnormal ReHo in the depressed group in this study, we performed further correlation analyses. We found a negative correlation between HAMD-17 scores and ReHo values in the right inferior frontal gyrus/right insula in the LORD group (*r* = −0.5778, *p* = 0.0120; Figure 3).

## Discussion

To our knowledge, this is the first to analyze and compare the regional neural activity of LORD and EORD based on rs-MRI technique using the ReHo method. The results of this study found that adult EORD and LORD have abnormal neuronal activity in some of the same brain regions at different ages. And relative to matched controls, different ReHo abnormalities were demonstrated at each age. Correlation analysis showed that ReHo values in the right inferior frontal gyrus/right insula were negatively correlated with clinical scales and could be used as a neuroimaging marker to distinguish LORD from EORD. This study provides new insights into functional brain activity in recurrent depression at different ages.

The results of this study showed that LORD had higher ReHo in the right fusiform gyrus/right middle temporal gyrus, left middle temporal gyrus/left angular gyrus, and right middle temporal gyrus/right angular gyrus compared with EORD. The concept of DMN was introduced by (Raichle et al., 2001) in 2001, which suggests that some brain areas are more active than others in a quiet, resting state without a task and are “activated,” while functional activity decreases when performing a task and becomes “negatively activated.” This region consists of the ventral medial prefrontal cortex, posterior cingulate/retosplenial cortex, lateral temporal cortex, dorsal medial prefrontal cortex, and inferior parietal lobules (including the angular gyrus) and is involved in the extraction of situational memory, detection of surroundings, and introspective states (Raichle et al., 2001; Raichle and Snyder, 2007; Buckner et al., 2008). The results of this study showed that most of the brain regions belonged to DMN. Most studies have regarded abnormal DMN function as a neurobiological marker of MDD (Gao et al., 2018; Goldstein-Piekarski et al., 2018; Sendi et al., 2021), DMN also changes in different stages of disease and at different ages, which is closely related to changes in clinical depression symptoms, and different therapeutic effects will also produce different responses to DMN (Huang et al., 2020; Pan et al., 2020). The middle temporal gyrus and angular gyrus are important constituent parts of the DMN (Buckner et al., 2008). The middle temporal gyrus not only participates in self-referential processing, but also plays an important role in autobiographical memory and regulates human emotional and mental activities (Sheldon et al., 2016). The angular gyrus is located at the junction of the parietal, temporal, and occipital lobes and is primarily involved in ontological functions such as semantic processing, number processing, attention, and memory (Seghier, 2013). Previous studies have shown abnormal DMN functional activity in patients with recurrent depression (Marchetti et al., 2012; Yan et al., 2019; Berwian et al., 2020). It was found that the recurrent depression group had hyperactivation in the right middle temporal gyrus compared to the HCs (Liu et al., 2017, 2019). Another study also found that the recurrent depression had abnormal bold signaling in the angular gyrus compared to the HCs (Yüksel et al., 2018). All of the above studies suggest abnormalities in the middle temporal gyrus and angular gyrus in patients with recurrent depression. The fusiform gyrus belongs to the visual cognitive network and together with the inferior temporal gyrus forms the ventral visual pathway, which is involved in the recognition of objects, words, and faces. The neural response of the fusiform gyrus increases with the intensity of the sad stimulus (Wu et al., 2011). ReHo was higher in the left superior temporal gyrus (Chen et al., 2012) and right fusiform gyrus (Shen et al., 2017) in late onset depression (LOD) compared with early onset depression (EOD), suggesting that compensatory elevation of the left superior temporal gyrus and right fusiform gyrus may have a pathogenetic mechanism for LOD. This is broadly consistent with the results of the present study. Therefore, the results of the present study suggest that ReHo is elevated in the right fusiform/right middle temporal gyrus, left middle temporal/left angular gyrus, and right middle temporal/right angular gyrus in LORD compared with EORD, and this partial compensatory elevation of DMN may be one of the differences in the brain mechanisms of recurrent depression in these two subtypes.

We also found that LORD had lower ReHo than EORD in the right inferior frontal gyrus/right insula and left superior temporal gyrus/left insula. The right inferior frontal gyrus, part of the ventral lateral prefrontal cortex, plays an important role in response inhibition and is primarily responsible for downregulating negative emotional responses and inhibiting unwanted information or inappropriate behavior (Kravitz et al., 2011). The insula is a cortical structure located deep in the brain and is involved in the processing of information related to emotion, attention, visceral sensation, etc., that is transmitted to the brain, as well as being involved in taste and vision (Sprengelmeyer et al., 2011). The insula cortex is connected to the frontal limbic area (Guo et al., 2015). Previous studies have found that metabolism in the insula cortex is enhanced in HCs when they recall sad events (Reiman et al., 1997). These two brain areas are closely related to the SN, which has been shown to be a functional brain network involved in perception, cognition, emotion, and social awareness (Menon and Uddin, 2010; Ham et al., 2013; Uddin, 2015; Chand and Dhamala, 2016; Touroutoglou et al., 2016). The SN has an important regulatory role for the DMN and the executive control network (ECN) (Touroutoglou et al., 2016). Previous, it was found that patients with recurrent depression had lower amplitude of low frequency fluctuations (ALFF) (Liu et al., 2017) and gray matter volume (Stratmann et al., 2014) in the right insula compared to HCs. It has also been found that middle-aged and older women with a history of depression have reduced FC between SN (right insula seed) and ECN (Vega et al., 2020). All of the above studies found that abnormalities in the function of the insula were closely associated with recurrent depression. Previous studies have found significantly enhanced ReHo in the right insula of LOD compared to old HCs, suggesting SN abnormalities in patients with LOD depression (Shen et al., 2017). Therefore, the results of this study suggest that there are differences in SN between LORD and EORD, which may also be the neuropathological pathogenesis of these two subtypes of recurrent depression. Further correlation analysis in this study showed that ReHo values in the right inferior insular frontal gyrus/right insula of the LORD group were negatively correlated with HAMD-17 scale scores. It is suggested that this region can be used as a neuroimaging marker with high sensitivity and specificity to distinguish between the two subtypes of recurrent depression. More research is needed in the future to clarify this point.

On the other hand, we also found that the ReHo in the left superior temporal gyrus/left insula were lower in the LORD compared to the EORD. The superior temporal gyrus is part of the auditory-verbal center and plays an important role in the processing of emotions, memory, and mental activity (Yao et al., 2018). Previous studies found that the LOD had higher ReHo values in the left superior temporal gyrus compared to the EOD, suggesting that compensatory elevation of ReHo in the left superior temporal gyrus may be the pathogenesis of LOD patients (Chen et al., 2012). Therefore, the results of this study suggest that the left superior temporal gyrus/left insula is also an important brain region in which differences exist between the two subgroups of patients with recurrent depression.

Interestingly, we observed that both the LORD and EROD groups together had increased ReHo in the left rolandic operculum gyrus/left superior temporal gyrus compared to HCs. The rolandic operculum gyrus and superior temporal gyrus belong to the auditory network (Oh et al., 2018; Koshiyama et al., 2020), which has the function of integrating auditory stimuli and processing them, and is closely related to human emotions (Phillips et al., 2001; Wang et al., 2019). Previous studies have found that depression scale scores are positively correlated with gray matter in the left rolandic operculum gyrus and left superior temporal gyrus, suggesting that these two brain regions also play a role in mood regulation (Besteher et al., 2017). One study showed that the insula is closely related to the operculum gyrus and that the insula/operculum gyrus is involved in interoception and interoceptive awareness, processes signals critical for self-awareness (Blefari et al., 2017). A meta-analysis showed that depressed patients had significantly increased activation of the right superior temporal gyrus during working memory (WM) compared to the HCs (Wang et al., 2015). In addition, abnormal fMRI connections between the right superior temporal lobe and subgenual cingulate cortex were also found in previous studies in patients with recurrent depression (Lythe et al., 2015). The above findings favorably support our observation of ReHo anomalies in the left rolandic operculum gyrus and left superior temporal gyrus. Therefore, these findings suggest that (Rotenstein et al., 2016) patients with recurrent depression may have features of auditory network dysfunction. (Rotenstein et al., 2016) ReHo abnormalities in the left rolandic operculum gyrus and left superior temporal gyrus may be important brain regions in distinguishing the recurrent depression group from the HCs, independent of age of onset.

However, there are still several limitations to this study. First, the small sample size of this study will affect the statistical validity to some extent, and a larger sample size is needed to confirm or overturn the current results. Second, only patients with recurrent depression were recruited in this study. Although there was a discontinuation period of at least 4 weeks, there may still be an influence of potential factors such as antidepressants. Finally, although there was no statistical difference in the frequency of recurrence between the two subtypes of depression groups, the study of first recurrence of depression in different age groups is more clinically relevant and further research in this area will be enhanced in the future.

## Conclusion

In conclusion, this study was based on rs-fMRI technique and used ReHo index analysis to initially explore the differences in regional neural activity between LORD and EORD. We found that although LORD and EORD had similar clinical symptoms, abnormal changes in neurological functional activity existed in some of the same brain regions, suggesting that different pathogenesis may exist in patients with different age onset. In addition, patients of each age group also exhibited different ReHo abnormalities relative to HCs.

## Data Availability Statement

The raw data supporting the conclusions of this article will be made available by the authors, without undue reservation.

## Ethics Statement

This study was reviewed and approved by the Ethics Committee of Guang’anmen Hospital, Chinese Academy of Traditional Chinese Medicine, China (NO. 2017-021-SQ). The patients/participants provided their written informed consent to participate in this study.

## Author Contributions

J-fS and L-mC drafted the manuscript and participated in data collection and analysis. J-kH, D-qG, C-lG, YM, YL, YH, and F-qX involved in data analysis and project design work. J-lF involved in the design of the experimental study and the revision of the manuscript. All authors contributed to the article and approved the submitted version.

## Funding

This research was supported by the Science and Technology Innovation Project of Chinese Academy of Traditional Chinese Medicine (CI2021A03301), and National Natural Science Foundation of China (82174282, 81774433), National Key Research and Development Program of China (2018YFC1705800).

## Conflict of Interest

The authors declare that the research was conducted in the absence of any commercial or financial relationships that could be construed as a potential conflict of interest.

## Publisher’s Note

All claims expressed in this article are solely those of the authors and do not necessarily represent those of their affiliated organizations, or those of the publisher, the editors and the reviewers. Any product that may be evaluated in this article, or claim that may be made by its manufacturer, is not guaranteed or endorsed by the publisher.

## Footnotes

^1^ http://www.rfmri.org/DPARSF
